# A potential role for somatostatin signaling in regulating retinal neurogenesis

**DOI:** 10.1038/s41598-021-90554-3

**Published:** 2021-05-26

**Authors:** Kurt Weir, Dong Won Kim, Seth Blackshaw

**Affiliations:** 1 Solomon H. Snyder Department of Neuroscience, Johns Hopkins University School of Medicine, Baltimore, MD 21205 USA; 2 Department of Ophthalmology, Johns Hopkins University School of Medicine, Baltimore, MD 21205 USA; 3 Department of Neurology, Johns Hopkins University School of Medicine, Baltimore, MD 21205 USA; 4 Institute for Cell Engineering, Johns Hopkins University School of Medicine, Baltimore, MD 21205 USA; 5 Kavli Neuroscience Discovery Institute, Johns Hopkins University School of Medicine, Baltimore, MD 21205 USA

**Keywords:** Developmental biology, Neuroscience

## Abstract

Neuropeptides have been reported to regulate progenitor proliferation and neurogenesis in the central nervous system. However, these studies have typically been conducted using pharmacological agents in ex vivo preparations, and in vivo evidence for their developmental function is generally lacking. Recent scRNA-Seq studies have identified multiple neuropeptides and their receptors as being selectively expressed in neurogenic progenitors of the embryonic mouse and human retina. This includes Sstr2, whose ligand somatostatin is transiently expressed by immature retinal ganglion cells. By analyzing retinal explants treated with selective ligands that target these receptors, we found that Sstr2-dependent somatostatin signaling induces a modest, dose-dependent inhibition of photoreceptor generation, while correspondingly increasing the relative fraction of primary progenitor cells. These effects were confirmed by scRNA-Seq analysis of retinal explants but abolished in *Sstr2*-deficient retinas. Although no changes in the relative fraction of primary progenitors or photoreceptor precursors were observed in *Sstr2*-deficient retinas in vivo, scRNA-Seq analysis demonstrated accelerated differentiation of neurogenic progenitors. We conclude that, while Sstr2 signaling may act to negatively regulate retinal neurogenesis in combination with other retinal ganglion cell-derived secreted factors such as Shh, it is dispensable for normal retinal development.

## Introduction

Neurotransmitters play important roles in regulating neurogenesis and development. Although neurotransmitters are generally considered in terms of neuronal signaling, neurotransmitter expression precedes the nervous system on both evolutionary and developmental time scales^1^. In fact, neurotransmitter genes are found in species without a central nervous system like sponges and the social amoeba *Dictysotelium*^2^. The receptors for several neurotransmitters such as GABA, glutamate, and serotonin are expressed on progenitor cells of the central nervous system prior to the formation of functional synapses^3–5^. The neurotransmitters themselves likely act as extrinsic signaling factors to guide neural development^1,3–5^.

It may be that developmental signaling represents the ancestral role for neurotransmitters before their use in signaling by mature neurons^2^. Neurotransmitters and neurotransmitter receptors are expressed in neural progenitors and/or immature neurons in many different regions of the vertebrate CNS^6–8^. Over the past three decades, there have also been a large number of studies in which pharmacological manipulation of a number of neurotransmitter systems have been reported to modulate multiple different aspects of neuronal development in different brain regions, including progenitor proliferation, neurogenesis, axonal guidance, synaptogenesis and neuronal survival^3,9,10^. The hippocampus is the brain region with the greatest number of reported neurotransmitter developmental signals: ATP, serotonin, VGF, vasoactive intestinal peptide (VIP), histamine, glycine, acetylcholine, dopamine, cholecystokinin, oxytocin, corticotropin-releasing hormone (CRH), ghrelin, nitric oxide (NO), GABA, and glutamate have all been found to play different roles in developmental and/or adult rodent hippocampal neurogenesis^3,6,11–22^. Other regions have fewer reported neurotransmitter developmental signals, like GABA, glutamate, histamine, VIP, and taurine^3,9,13,15^ in neocortical development, and pituitary adenylate cyclase-activating peptide (PACAP) in cerebellar development. Serotonin regulates the development of multiple brain regions. However, the specificity of these ligands–particularly at the high concentrations often used in these studies–is unclear. Although mutants for many genes directly related to neurotransmission are available, developmental phenotypes are seldom characterized. The precise role of neurotransmitters in regulating brain development thus remains largely unclear.

The retina is an accessible and relatively less complex CNS region that serves as a useful model for understanding molecular mechanisms controlling the development of more complex brain regions^23^. Extrinsic signaling also plays a critical role in regulating retinal development. In addition to classical growth and differentiation factors that are present in the developing retina–such as bFGF and TGF alpha^24^, GDF11^25^, VEGF^26^ and SHH^27^–a number of neurotransmitters are expressed by retinal progenitors and/or immature retinal neurons^28^. Pharmacological manipulation of serotonin, glutamate, or GABA signaling impacts retinal development in *Xenopus laevis*^1^, and endogenous opiate-like peptides have been reported to regulate neurogenesis in rodent retina^27,29^. However, genetic data to support these findings is lacking. Moreover, until recently, comprehensive and quantitative analysis of the cellular expression patterns of neurotransmitter biosynthetic enzymes and receptors during retinal development has not been available. This has changed with the generation of several large-scale single-cell RNA-sequencing (scRNA-Seq) datasets from developing mouse and human retina^30,31^.

This work has identified four different neuropeptides and/or their receptors as being strongly and selectively expressed during early stages of retinal development—somatostatin, neuropeptide Y, galanin, and proenkephalin. Each of these neuropeptides has been reported to regulate brain development. Somatostatin acts as a generalized growth factor inhibitor, and somatostatin expression in the hypothalamus decreases with age along with hypothalamic neurogenesis^13,32^. Neuropeptide Y promotes proliferation in the subventricular zone and hippocampal precursor cells^6,13,15,33^. It also promotes proliferation of dissociated rat retinal progenitor cells, apparently through NO^34^. Galanin, acting through its receptor GalR3, promotes neurogenesis in the adult hippocampus^6,13,17,35^. Finally, proenkephalin impacts cerebellar development and adult hippocampal neurogenesis^36,37^.

To define any of these neuropeptides as an endogenous regulator of retinal development, we need to identify (1) a source of the ligand, (2) impacts on neurogenesis from activation and knockout of the appropriate receptors, and (3) an in vivo phenotype ^38^. To accomplish this, we use publicly-available single-cell RNA-seq data of mouse and human retinal development^30,31^, a pharmacologic screen on murine embryonic retinal explants, and single-cell sequencing of retinal explants and mutant retinas. We find that though they play roles in development elsewhere in the CNS or in other in vitro situations, neuropeptide Y, galanin, and proenkephalin do not impact neurogenesis in embryonic retinal explants. Activation of the somatostatin receptor *Sstr2* reduces the production of photoreceptors in this model. However, somatostatin likely plays a redundant role in retinal development as its knockout does not produce the same effect in vivo.

## Results

### ScRNA-seq identifies neuropeptides dynamically expressed during retinal neurogenesis

A previously published single-cell RNA sequencing (scRNA-seq) study profiled mouse retinal development across ten-time points. We reasoned that this gene expression dataset could be used to identify candidate neurotransmitters impacting mouse retinal development. We downloaded the dataset and used the stored cell type and age data to investigate the expression of neuropeptides in mouse development.

**Figure 1:**
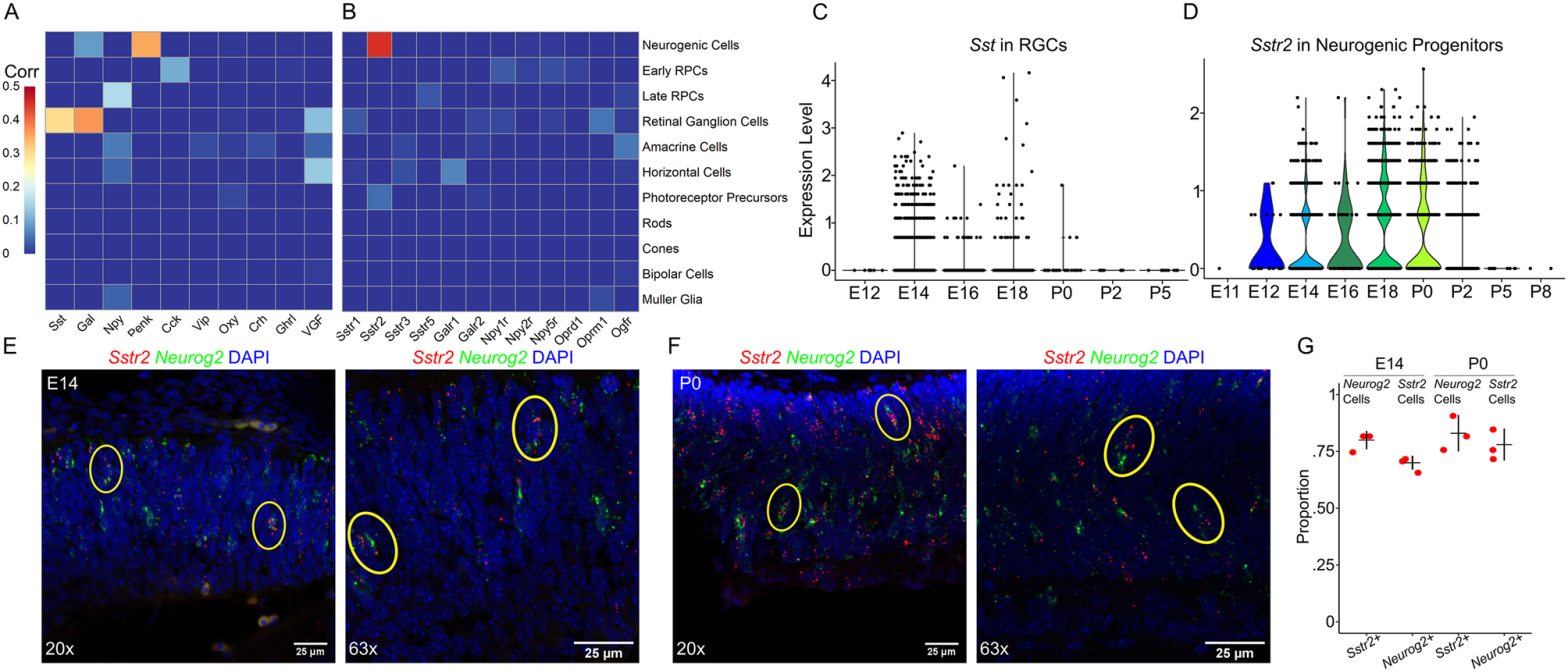
Cell-specific expression of neuropeptides and neuropeptide receptors in developing mouse retina. (**A**) Heatmap of the correlation of expression of a neuropeptide with a given cell type across the full course of mouse retinal neurogenesis (E11-P14)^31^. (**B**) Correlation of expression of a neuropeptide receptor with a given cell type across the full course of mouse retinal neurogenesis. *Sstr4*, *Galr3*, and *Npy4r* were not expressed at detectable levels in the dataset. (**C**) Violin plot of *Sst* expression in retinal ganglion cells across mouse development. Each point represents a single cell. Expression determined by the SCT method in Seurat. (**D**) Violin plot of *Sstr2* expression in neurogenic progenitor cells across mouse development. (**E**) E14 and (**F**) P0 mouse retina hybridized with RNAscope smFISH probes for *Sstr2* (red) and *Neurog2* (green) and counterstained with DAPI (blue). Example cells expressing both *Sstr2* and *Neurog2* circled in yellow. 20 × Resolution on the left and 63 × resolution on the right. Scale bars represent 25 μm. (**G**) Dot plot quantifying overlap of cellular expression for *Sstr2* and *Neurog2* at E14 and P0. Black crosses represent average and standard deviation. N = 3 retinas for each age. Correlation calculated using base R function ‘cor()’.

Cellular expression patterns of neuropeptides are readily identified using scRNA-seq. The neuropeptides somatostatin (Sst), galanin (Gal), neuropeptide Y (Npy), and proenkephalin (Penk) were selected for further study as they showed the greatest correlation of expression with a single cell type (Fig. 1A). *Sst* and *Gal* are expressed by mouse retinal ganglion cells most strongly between E14 and P0 (Fig. 1A,C, S1A), and by human retinal ganglion cells between gestational day 42 and gestational week 13 (Fig. S1D,F). Gal is also strongly expressed in early-stage neurogenic progenitors^39^. *Npy* is expressed primarily by late-stage primary retinal progenitor cells between E18 and P2, and *Penk* is expressed by neurogenic progenitor cells mostly between E14 and P2 (Fig. 1A, S1B,C). The expression of neuropeptides by specific cell types over delimited windows during retinal development suggests that they may be acting as signaling molecules impacting retinogenesis.

In sharp contrast, with one exception, the receptors for Sst, Gal, Npy, and Penk were either not detected or only barely detectable in both developing mouse and human retina. The one notable exception was somatostatin receptor 2 (*Sstr2*) which is strongly and selectively expressed by neurogenic RPCs from E12 to P2 in mice (Fig. 1B,D). This matches the period when *Sst* is prominently expressed in immature RGCs. *SSTR2* is also expressed by neurogenic RPCs, and undifferentiated cones, bipolar cells, and RGCs, in the human retina, while *SST* is also expressed in immature RGCs (Figure S1D-G). To confirm the expression of *Sstr2* in the neurogenic progenitors of developing mouse retina, we used RNAscope single molecule fluorescent in situ hybridization to probe for *Sstr2* and *Neurog2* mRNA expression in E14 and P0 retinas (Fig. 1E,F). We found a high level of overlap in expression with a mean of 70% (+/− 3%) of *Sstr2* + cells expressing *Neurog2* and 80% (+/− 4%) of *Neurog2* + cells expressing *Sstr2* at E14, while at P0, 78% (+/− 7%) of *Sstr2* + cells expressed *Neurog2* and 83% (+/− 8%) of *Neurog2* + cells expressed *Sstr2* (Fig. 1G). This led us to hypothesize that Sst could be acting as a signal released by retinal ganglion cells to influence neurogenic progenitors through Sstr2.

### Sstr2 signaling inhibits expression of photoreceptor marker genes in retinal explants

We decided to use a pharmacologic screen, treating mouse embryonic retinal explants with small molecule agonists and antagonists to the receptors of the four neuropeptides of interest, as a cost-effective test for an effect on retinal development. Because *Sstr2* was highly and selectively expressed in neurogenic RPCs, while other Sst receptors were not expressed or barely detectable, we used an Sstr2-specific agonist and antagonist. For the other candidates, we ordered the neuropeptides themselves and antagonists general to all of their receptors; except Npy, for which no general antagonist was available. The explants were cultured for a period roughly equivalent to the expression of the relevant neuropeptide in vivo with either agonist, antagonist, or solvent control. We used qRT-PCR to measure the relative levels of multiple cell type marker genes between treated retinas and control as a readout of the effects of the treatments on the expression of cell type-specific markers.

**Figure 2:**
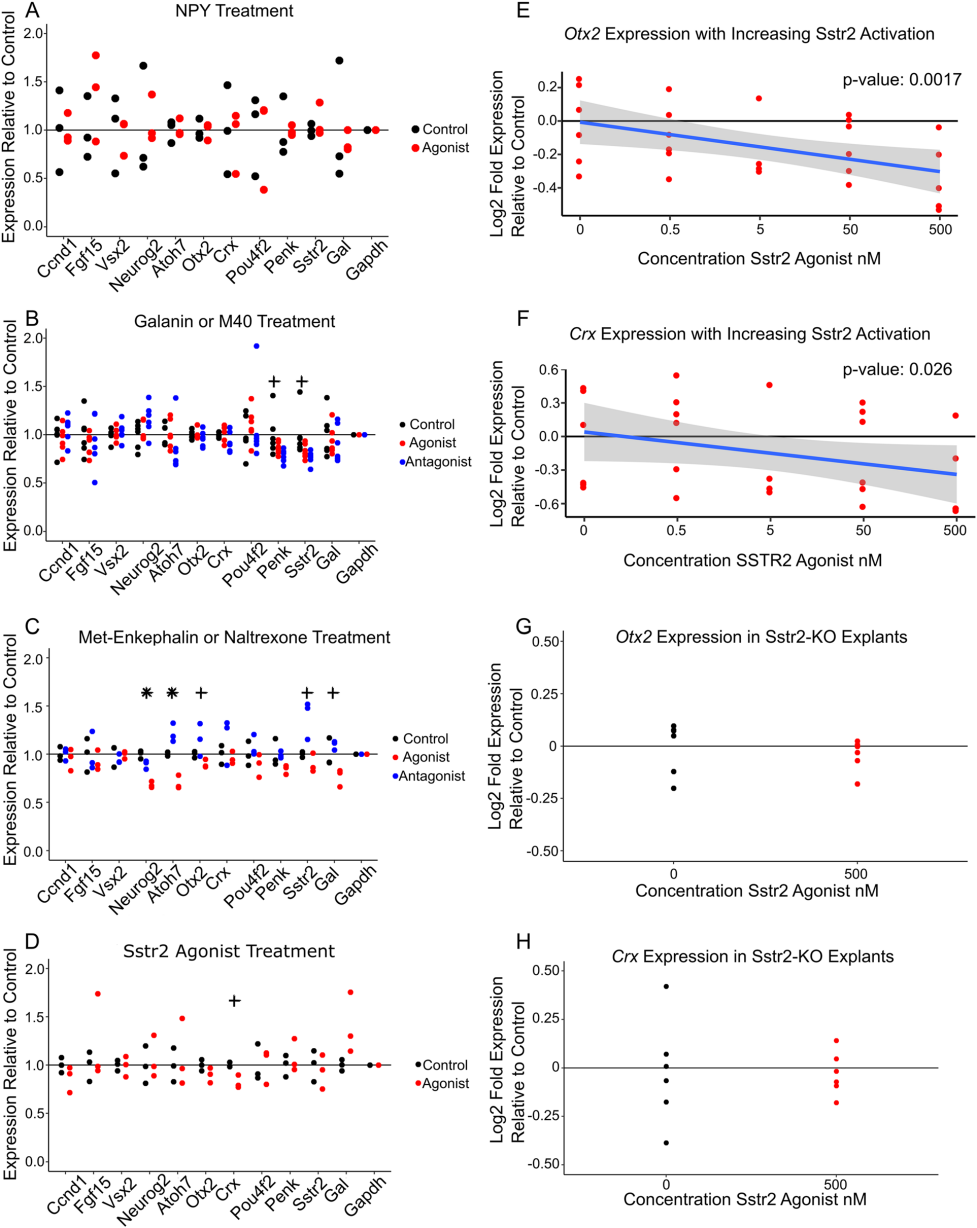
Analysis of cell type-specific markers by qRT-PCR in embryonic mouse retinal explants treated with neuropeptide agonists and antagonists. Cell type marker genes tested include *Ccnd1* (all progenitors); *Fgf15* and *Vsx2* (primary progenitors); *Neurog2* and *Atoh7* (neurogenic progenitors); *Otx2* and *Crx* (photoreceptor precursors); *Pou4f2* (retinal ganglion cells); *Penk*, *Sstr2*, and *Gal* (neuropeptides in the study, with each of these also being selective markers of neurogenic progenitors); and *Gapdh* (internal control). Each point represents a sample (average of 3 technical replicates). Each sample was tested for each gene. (**A**) Marker gene expression relative to average control with Npy treatment. Concentration = 1 μM. n = 3 each condition. Explants treated E18-P2. (**B**) Marker gene expression relative to average control with Galanin or M40 treatment. Concentration = 1 μM. n = 6 each condition. Explants treated E14-P0. (**C**) Marker gene expression relative to average control with Met-Enkephalin or Naltrexone treatment. Concentration = 500 nM. n = 3 each condition. Explants treated E14-P0. (**D**) Marker gene expression relative to average control with treatment with SSTR2 agonist (1R,1'S,3'R/1R,1'R,3'S)-L-054,264. Concentration: 500 nM. n = 3 each condition. Explants treated E14-P0. (**E**) *Otx2* expression relative to average control of explants treated with increasing levels of Sstr2 agonist. The trend line represents a linear model, with gray area denoting the 95% confidence interval. n = 6 each condition (except for the 5 nM treatment, where n = 4). Explants treated between E14-P0. (**F**) *Crx* expression relative to average control of explants treated with increasing concentrations of Sstr2 agonist. Trend line represents a linear model with the gray area denoting 95% confidence interval. n = 6 each condition (except for 5 nM treatment, where n = 4). Explants treated from E14-P0. (**G)** *Otx2* expression relative to average control of *Sstr2*^−/−^ explants treated with 500 nM Sstr2 agonist. n = 6 each condition. (**H**) *Crx* expression relative to controls in *Sstr2*^−/−^ explants treated with 500 nM Sstr2 agonists. n = 6 each condition. Explants treated E14-P0. (**A**–**D**) *P*-value calculated using ANOVA. + indicates a nominally significant *P*-value below 0.05. *Indicates a *P*-value significant at a Bonferroni correction below 0.0045. (**E**, **F**) *P*-value calculated with linear model of expression ~ condition + RNA extraction batch. (**G**, **H**) *P*-value calculated using student’s t-test.

Treatment with Npy, galanin, and the galanin receptor antagonist M40 did not produce significant changes in expression for the tested marker genes (Fig. 2A,B). Treatment with Met5-enkephalin, a bioactive processed product of Penk, decreased expression of the neurogenic progenitor markers *Atoh7*, *Neurog2*, and *Sstr2* while the broad-spectrum opiate receptor antagonist naltrexone increased expression for *Atoh7* and *Sstr2* as measured by ANOVA (*P*-value < 0.0045, Fig. 2C). These results suggest that Penk signaling may reduce the proportion of neurogenic progenitor cells in retinal explants. Meanwhile, treatment with the Sstr2-specific agonist (1R,1'S,3'R/1R,1'R,3'S)-L-054,264 consistently nominally significantly decreased expression of the photoreceptor markers *Crx* and *Otx2* (*P*-value < 0.05, Fig. 2D, S2B). Treatment with the Sstr2-specific antagonist CYN 154,806 did not impact marker gene expression, possibly because retinal explants lack RGCs, the endogenous source of Sst (Fig. S2A). This similarly suggests that Sst signaling through Sstr2 negatively regulates photoreceptor differentiation.

We next conducted further studies of the potential role of Penk and Sst/Sstr2 in regulating retinal development. To increase our ability to detect an impact on retinal development, the initial assay had been performed using concentrations orders of magnitude in excess of the ligand’s reported EC50 or IC50 values. It is therefore possible that the observed effects were due to nonspecific or off-target effects of these ligands, rather than selective modulation of neuropeptide receptors. In order to test this, we cultured explants in a range of treatment concentrations below that tested previously. We then tested for expression of *Atoh7*, *Neurog2*, and *Sstr2* following Met5-enkephalin and naltrexone treatment, and for *Crx* and *Otx2* following treatment with the Sstr2 agonist. Treating with reduced concentrations of Met5-enkephalin and naltrexone failed to reproduce the effect seen at saturating concentrations (Fig. S2C-H). However, treatment with lower concentrations of the Sstr2 agonist reduced photoreceptor marker gene expression in a dose-dependent manner, suggesting that it was indeed acting through a specific receptor (*P* < 0.05, Fig. 2E,F). Moreover, treatment with 500 nM Sstr2 agonist had no significant effect (*P* > 0.1) on *Crx* and *Otx2* expression in explants obtained from *Sstr2*^*−/−*^ mice, as measured by t-test (Fig. 2G,H), demonstrating that these effects are selectively mediated by activation of Sstr2 signaling.

### Sstr2 signaling inhibits photoreceptor specification in embryonic retinal explants in a dose-dependent manner

**Figure 3:**
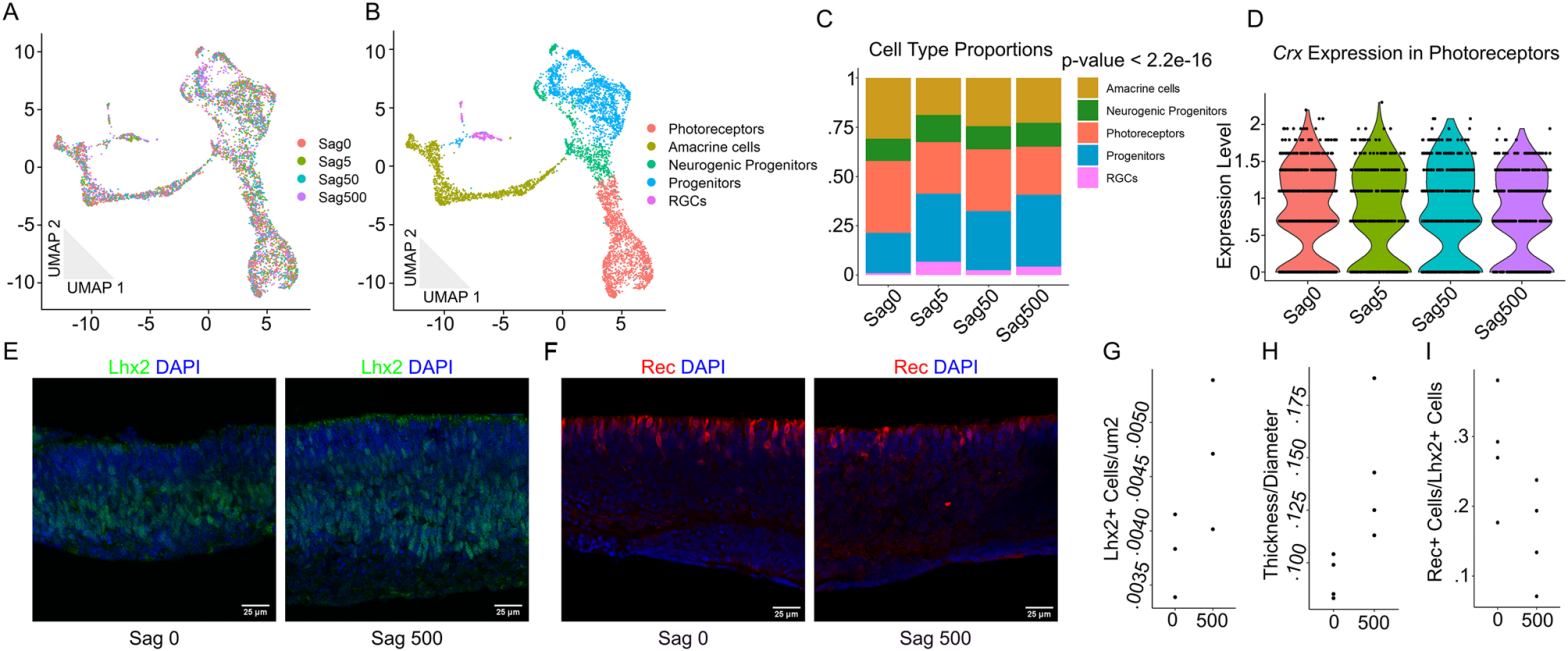
MULTI-seq and immunohistochemistry analysis of explants treated with four different concentrations of Sstr2 agonists reveals changing cell type proportions with increasing treatment. Four concentrations tested: 0, 5 nM, 50 nM, 500 nM Sstr2 agonist. n = 2 samples per condition. n = 5,607 cells (Sag0: 1556, Sag5: 1277, Sag50: 1204, Sag500: 1570). (**A**) 2D UMAP dimension reduction representation of the pooled explant cells colored by treatment condition. (**B**) Cells colored by identified cell type. (**C**) Bar graph representation of the proportion of each cell type within each condition. *P*-value for significance in difference of cell type proportions across treatment conditions were calculated using Pearson’s Chi-squared test. (**D**) Violin plot of expression of *Crx* in photoreceptors for each condition. Was not found to be significantly different by Seurat function ‘FindAllMarkers’. (**E**–**I**) Retinal explants stained with (**E**) Lhx2 (green) or (**F**) Recoverin (red) antibodies and DAPI (blue) counterstaining after treatment with 0 nM Sstr2 agonist (left panel) or 500 nM Sstr2 agonist (right panel). Scale bars represent 25 μm. Dot plots representing (**G**) the density of Lhx2 + cells (n = 3, one replicate per condition were lost during staining), (**H**) ratio of explant thickness to diameter (n = 4), and (**I**) the density of Rec + cells corrected by the average density of Lhx2 + cells for that condition (n = 4). Differences were not found to be significant by Welch’s two-sample t-test.

It is unclear whether Sstr2 signaling actually inhibits photoreceptor generation or reduces expression levels of *Otx2* and *Crx*. While qPCR cannot distinguish between these possibilities, scRNA-seq can readily do so. We applied MULTI-seq^40^ to retinal explants grown in a range of Sstr2 agonist concentrations. MULTI-seq allows us to generate a single expression library with cells labeled for each treatment condition (Fig. 3A). After clustering and cluster marker identification, we identified the cell type corresponding to each cluster (Fig. 3B, S3A). With the sample and cell type identity known for each cell, we quantified and compared the proportions of cell types across the different treatment conditions (Fig. 3C). Increasing the concentration of Sstr2 agonists decreased the proportion of photoreceptors and increased the proportion of primary progenitors as a fraction of cells in the explants. Cell type proportions across different treatment conditions were significantly different by chi-squared test (*P* < 2.2 E-16). In addition, the level of photoreceptor marker gene expression in photoreceptor clusters was not significantly changed in response to different agonist concentrations by Wilcoxon rank-sum test (Fig. 3D, S3B). We confirmed the differences in cell type proportions by immunostaining for the primary progenitor marker gene Lhx2, the cell cycle marker Ki67, and the photoreceptor marker Recoverin. Treatment with saturating levels of Sstr2 agonist increased the relative fraction of Lhx2 + cells and the thickness of the explants, while decreasing the relative ratio of Recoverin + cells to Lhx2 + cells (Fig. 3E–I). Though suggestive, these differences did not reach significance by Welch’s t-test. This demonstrates that somatostatin signaling exerts, at best, modest changes in retinal cell type composition in retinal explants. The treatment did not impact the area-normalized number of cells expressing Ki67, though the observed increase in explant thickness may have affected this measurement (Fig. S3C,D). While not statistically significant, the immunostaining results are in agreement with both the qPCR and MULTI-seq data. The qPCR, MULTI-seq, and immunostaining results taken together demonstrate that, in retinal explants, Sstr2 signaling modestly inhibits photoreceptor specification while increasing the relative fraction of primary RPCs.

### No significant change in cell composition is detected in ***Sstr2***^***−/***−^ retinas

**Figure 4:**
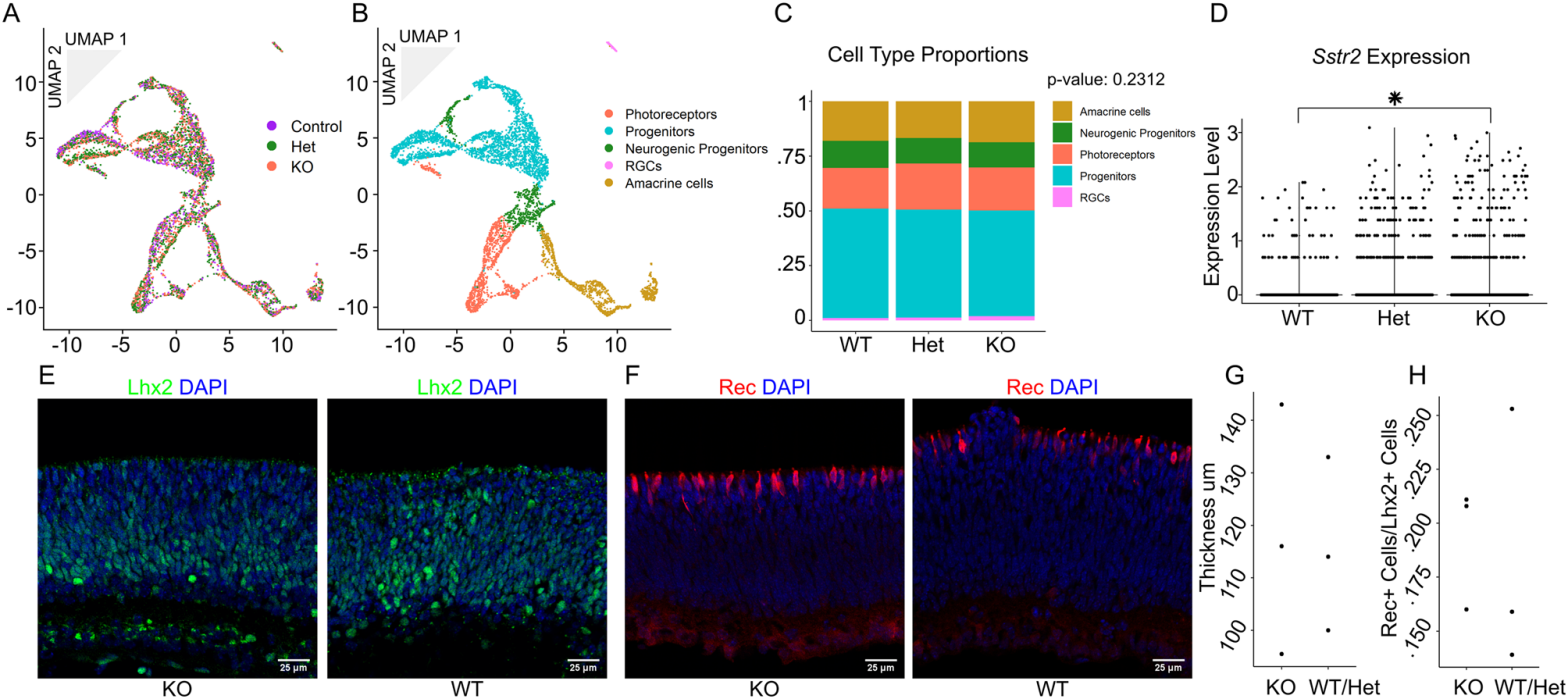
MULTI-seq and immunohistochemistry analysis of wildtype, *Sstr2*^+/−^, and *Sstr2*^−/−^ littermates reveals no change in cell-type proportions at P0. n = 2 samples per genotype (except + / + where n = 1). n = 5,706 cells (*Sstr2*^+*/*+^: 1207, *Sstr2*^+/−^: 2341, *Sstr2*^−/−^: 2158). (**A**) 2D UMAP dimension reduction representation of the pooled retinal cells colored by genotype. (**B**) Cells colored by identified cell types. (**C**) Bar graph representation of the proportion of each cell type within each genotype. *P*-value for significance in difference of cell type proportions across genotypes were calculated using Pearson’s Chi-squared test. (**D**) Violin plot of expression of *Sstr2* in cells for each condition. Was found to be significantly different by Seurat function ‘FindMarkers’. (**E**–**H**) P0 Retinas stained with (**E**) Lhx2 (green) or (**F**) Recoverin (red) antibodies and counterstained with DAPI (blue) with *Sstr2*^−/−^ in the left panel and *Sstr2*^+/+^ in the right panel. Scale bars represent 25 μm. Dot plots representing (**G**) retinal thickness (n = 3) and (**H**) the density of Rec + cells corrected by the density of Lhx2 + cells for that biological replicate (n = 3). No significant difference found by Welch’s two-sample t-test.

To test whether Sstr2 signaling regulates photoreceptor differentiation in vivo, we applied MULTI-seq to the retinas of littermates that were wild type, heterozygous, and homozygous for a targeted deletion of *Sstr2*. We hypothesized that KO mice would have greater proportions of photoreceptors than their littermates. Mice were collected at P0 to match the age at which the explants were tested and P14 to determine whether terminal differentiation and/or survival of specific retinal cell types were affected. Following the same procedure for the sample and cell-type identification as we used for the explants (Fig. 4A,B, S4A,E–G), we found no significant difference in cell-type proportions between littermates of different genotypes at P0 by chi-squared test (Fig. 4C). The genotypes at P14 had significantly different cell type proportions, but this was likely driven by the over enrichment of rods in the Het cells compared to both WT and KO genotypes (Fig. S4H). While the significance of this is unclear, the smaller total number of cells profiled in each sample in the P14 dataset may have led to greater variability in the observed cell type proportions, leading to the observed discrepancy. We confirmed the similarity in cell-type proportions across genotypes by immunostaining for Lhx2, Ki67, and Recoverin, which showed no difference in retinal thickness, the density of Lhx2 + or Ki67 + cells, or in the ratio of Recoverin + to Lhx2 + cells by Welch’s t-test at P0 (Fig. 4E–H, S4B–D). There was also no difference in Lhx2 + cell density or the combined thickness of the outer segment and outer nuclear layer at P14, or in the total retinal area controlled for body length in the adult mouse as measured by ANOVA (Fig S5A-E). All results found in the *Sstr2* KO mouse by immunostaining were recapitulated in the *Sst* KO mouse (Fig. S6A-K). However, *Sstr2* mRNA levels were increased in neurogenic RPCs in P0 *Sstr2* mutants relative to controls as measured by Wilcoxon rank-sum test (*P*-value = 3.35E-5) (Fig. 4D), implying that Sstr2 signaling may negatively regulate *Sstr2* transcription.

### Sstr2 signaling promotes retinal neurogenesis and inhibits expression of progenitor-specific genes in retinal explants

We next examined our scRNA-Seq data in more detail to determine which genes showed altered expression in *Sstr2*^*−/−*^ retinas. We hypothesized that though we did not see a change in cell-type proportions in vivo that were complementary to those seen in agonist-treated ex vivo retinal explants*,* we might nonetheless observe complementary changes in gene expression. We compared differentially expressed genes of wild type vs. *Sstr2*^*−/−*^ neurogenic RPCs in vivo and 500 nM versus 0 nM Sstr2 agonist-treated neurogenic cells ex vivo. We identified only one differentially expressed gene in common between these two comparisons. This was *Xist*, and likely simply reflected differences in the sexes of the animals among the different samples. In total, only eleven genes were identified in vivo and two identified ex vivo by Wilcoxon rank-sum test (*P*-value < 5E-5) (Suppl. Tables 1, 2). Neither of these lists of genes suggested a clear transcriptional impact from Sstr2 perturbation. We felt that traditional gene expression tests may be underpowered when looking for modest changes in gene expression in single cell types.

**Figure 5:**
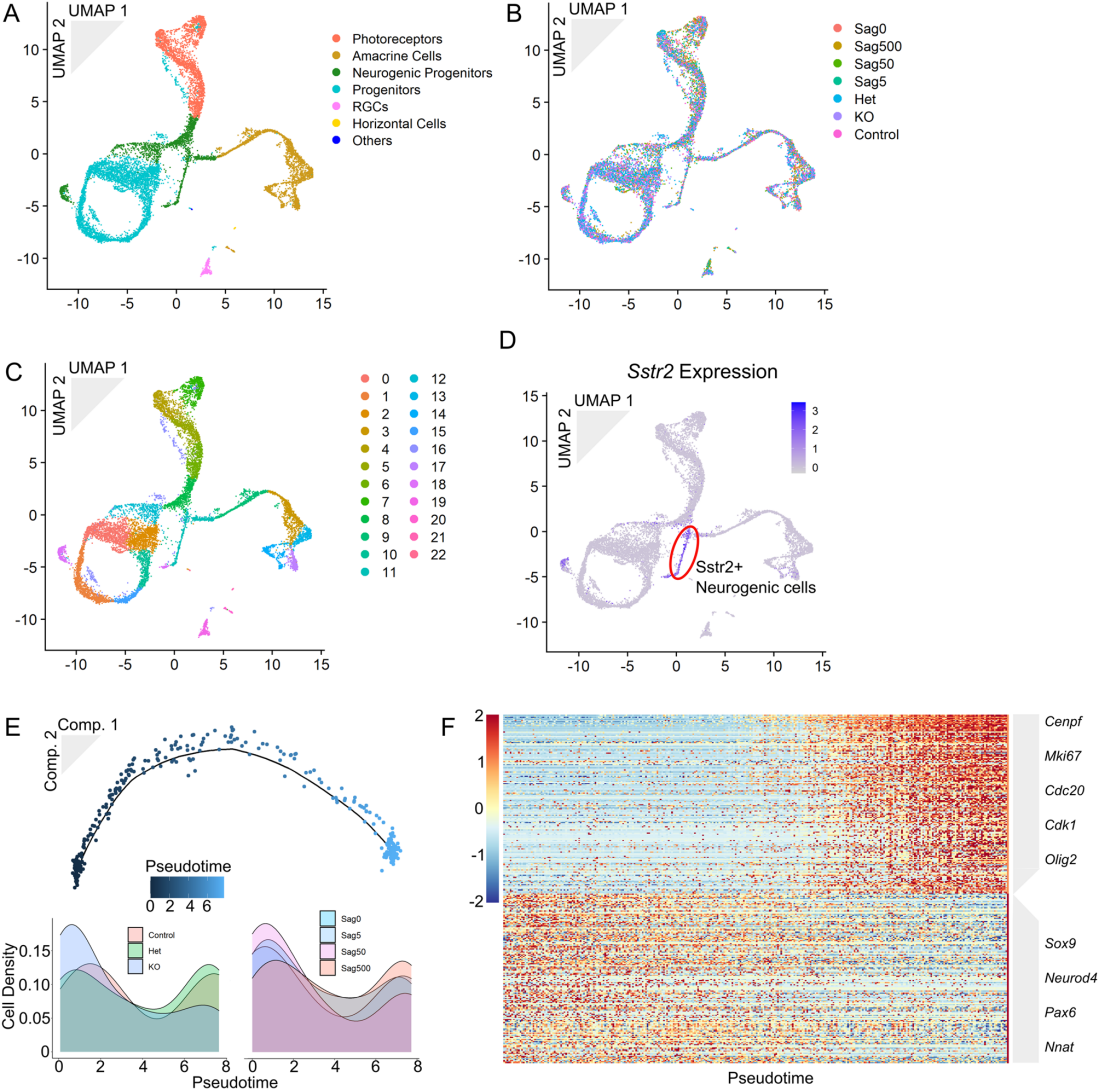
Integrated dataset of explant and retina cells reveals shared genetic changes. The explant and P0 datasets were integrated following the Seurat protocol. n = 11,313 cells. (**A**) 2D UMAP dimension reduction representation of the integrated datasets colored by identified cell type. (**B**) Cells colored by genotype or treatment condition. (**C**) Cells colored by Seurat-identified cluster. (**D**) Cells colored by *Sstr2* expression. (**E**) Monocle-generated trajectory of *Sstr2*-expressing neurogenic cells from cluster 11 (top). Density plot for cells of each in vivo genotype aligned along pseudotime (bottom left). Density plot for cells of each ex vivo treatment aligned along pseudotime (bottom left). (**F**) Heatmap of two clusters of genes that either increase with pseudotime (top 170 genes) or decrease with pseudotime (bottom 161 genes). Genes identified with Monocle 2 function ‘differentialGeneTest()’, q-value cutoff 0.05. Genes clustered by k-means (seed = 37).

We then looked for a mechanism of action for Sstr2 signaling by testing whether genes that differ between genotypes in vivo could distinguish cells from different treatments ex vivo, as a more nuanced way to evaluate differences in gene expression. To do this, we integrated our ex vivo and in vivo datasets (Fig. 5A,B) and, reasoning that the neurogenic cells that express *Sstr2* are the most likely to be affected by differences in Sstr2 signaling, selected neurogenic cells that express *Sstr2* from cluster 11 (Fig. 5C,D). We then used the 58 genes that are differentially expressed between *Sstr2*-positive neurogenic RPCs from wildtype, *Sstr2*^+*/−*^, and *Sstr2*^*−/−*^ mice to guide the pseudotime program Monocle 2^41^ in aligning all of the Sstr2-positive neurogenic cells along a pseudotime trajectory (Fig. 5E, Suppl. Table 3). This analysis identified an unbranched trajectory. While high Sstr2-activated (wild type and heterozygous retinal, and 500 nM-treated explant) cells were distributed equally along this trajectory, low Sstr2-activated (*Sstr2*^*−/−*^ retinal, and 0, 5, and 50 nM-treated explant) cells were significantly comparatively enriched at lower pseudotime values (one-sided KS test, p value = 0.0054). None of the samples combined in the low or high groups had significantly different pseudotime distributions from each other as measured by a two-sided KS test. These results led us to conclude that Sstr2-related perturbations created some shared differences across the two systems.

We next tested for genes that changed in expression across our pseudotime trajectory to see if we could resolve which genes are impacted most by Sstr2 signaling. We identified 331 genes, 170 of which increased with pseudotime and Sstr2 signaling, and 161 of which decreased by likelihood ratio test (q-value < 0.05, Fig. 5F, Suppl. Tables 4, 5). The online gene set enrichment tool DAVID^42^ showed a strong enrichment for cell-cycle genes among the genes that increased with pseudotime, including *Cdc20*, *Cenpf*, and *Mki67*. Genes that regulate neuronal development and differentiation showed decreased expression with pseudotime, including *Neurod4*, *Nnat*, and *Sox9*. It appears that the pseudotime trajectory captures the transition from proliferative neurogenic RPCs (high pseudotime values) to differentiating post-mitotic neural precursors (low pseudotime values) and that genotypes and conditions with no or low Sstr2 signaling lead to increased cell cycle exit and neurogenesis. In combination with our results obtained from retinal explants, where increasing Sstr2 activation reduced photoreceptor generation, we conclude that Sstr2 signaling modestly inhibits retinal neurogenesis in retinal explants.

Because of the relatively modest impact of Sst/Sstr2 signaling on retinal neurogenesis, we hypothesize that additional signals may be released by RGCs to modulate retinal neurogenesis. To investigate this possibility further, we again analyzed the publicly available scRNA-Seq mouse retinal development dataset. Mouse RGCs express the neuropeptide Pacap and signaling molecule Sonic Hedgehog (Shh) during the same interval that *Sst* and *Sstr2* are expressed (Fig. S7A,C,E). When examining the full course of mouse retinal development, neither the Shh receptor *Ptch1* nor the Pacap receptor *Vipr2* are specifically expressed in neurogenic RPCs, though they are specifically expressed in late-stage primary RPCs and photoreceptor precursors, respectively (Fig. S7A). Additionally, both genes are coexpressed with *Sstr2* in neurogenic RPCs (Fig. S7D,F). When examining the full course of human retinal development, both *Pacap*, and *Shh* are more strongly expressed in Müller glia than RGCs (Fig. S7B), although both are co-expressed with *Sst* in RGCs (Fig. S7C,E). Both *Ptch1* and *Vipr2* are co-expressed in neurogenic RPCs and late-stage primary RPCs in developing human retina, while *Vipr2* is also expressed in early-stage RPCs and RGCs (Fig. S7B). As in mouse, both *Ptch1* and *Vipr2* are coexpressed with *Sstr2* in neurogenic RPCs (Fig. S7D,F). Pacap and Shh may therefore act in combination with Sst as potentially redundant signals that modulate retinal neurogenesis.

## Discussion

We identified four neuropeptides that are transiently, strongly, and specifically expressed during mouse retinal development by analyzing scRNA-seq data obtained from developing mouse retina. We tested whether they regulate retinal development using a combination of small-molecule ligands and qRT-PCR analysis of major retinal cell type markers in embryonic retinal explants. Agonists and antagonists of these neuropeptides—Npy, Gal, and Penk—did not produce significant dose-dependent changes in any retinal cell type markers tested. These results agree with Brodie-Kommit et al., who found no effect on RGC density in Gal-deficient mice^39^. However, agonist-dependent activation of the Sst receptor Sstr2 inhibited photoreceptor generation and led to a compensatory increase in the proportion of primary progenitors. The specificity of these effects was confirmed by their loss in *Sstr2*-deficient retinal explants. However, while Sstr2 signaling exerts a modest inhibitory effect on photoreceptor specification in retinal explants, this was not seen in vivo, with relative numbers of major retinal cell types unchanged in the *Sstr2* mutant relative to wild type. This could reflect differences in the signaling mechanisms downstream of Sstr2 in explants compared to intact retinas that result from stresses associated with explant culture. ScRNA-Seq analysis revealed that retinal explants treated with Sstr2 agonists showed changes in gene expression in neurogenic RPCs that were complementary to those seen in *Sstr2* mutants. These gene expression changes suggest that RGCs may secrete Sst as a quorum signal to negatively regulate retinal neurogenesis. However, the lack of impact on cell type proportion in intact retinas also suggests that Sstr2 signaling is dispensable for regulation of photoreceptor specification in vivo.

Though *Sstr2* KO did not affect retinal cell type proportions in vivo, similar effects to what we observe in Sstr2-treated explants have been reported following manipulation of retinal Sonic Hedgehog (Shh) signaling. Wang et al. found that ablation of embryonic RGC-derived Shh signaling in the mouse retina results in precocious cell cycle exit and differentiation of progenitor cells. This resulted in an accelerated generation of photoreceptors, depletion of the progenitor pool, and reductions in the proportions of later-born cell types^43^. This matches the phenotype seen in our embryonic retinal explants where treatment with an Sstr2-specific agonist resulted in reduced photoreceptor proportion and an increase in the number of primary progenitors. Wang et al. also found that ablation of Shh signaling resulted in a decrease of *Ccnd1* and *Hes1* gene expression, while RPC-enriched genes such as *Hes2* and *Ccnb1* and other cell cycle genes were also decreased upon disruption of Sstr2 signaling. Though we did not observe changes in cell composition in *Sstr2* mutant retinas in vivo, pseudotime analysis revealed that neurogenic progenitors in these mutants were relatively more mature than controls, while neurogenic progenitors were relatively less mature in explants treated with Sstr2 agonists. Sstr2 + neurogenic cells in both intact *Sstr2*^*−/−*^ mutant retina and in explants treated with no or low levels of Sstr2 agonist showed increased expression of genes associated with terminal differentiation relative to wildtype, *Sstr2*^+*/−*^, or Sstr2-expressing neurogenic cells in retinal explants that were treated with high levels of Sstr2 agonist, which in turn showed higher expression of genes enriched in retinal progenitors. Sstr2 signaling may therefore work in tandem with Shh signaling to prevent maturation and differentiation of neurogenic progenitor cells.

A similar dependence on context for the impact of signaling on retinal cell type proportions was reported by Pearson and colleagues^44^. They found that an agonist for the ATP receptor P2RY2 only impacted chick retinal explant development in the absence of the RPE, the endogenous source for ATP in chick retinogenesis. The retinal explants used in the current study lack viable RGCs – the endogenous source of secreted Sst and Shh at this age. We suggest that in the developing retina, RGCs release multiple redundant signals to inhibit cell cycle exit and differentiation in neurogenic progenitors. When retinas are grown as explants, all of the signals are lost, allowing a single exogenous agonist to influence the fate of retinal progenitors. This would explain why Sstr2-mediated signaling regulates cell type proportions ex vivo but not in vivo.

Interestingly, the proportion of neurogenic progenitors remained consistent across explants at each concentration of Sstr2 agonist, even though fewer cells underwent neurogenesis in explants treated with higher concentrations of agonist. The transition of primary progenitor cells to neurogenic progenitors may therefore be inhibited by the presence of a high concentration of neurogenic cells. If fewer neurogenic cells are exiting mitosis to generate neurons, fewer primary progenitors are entering a neurogenic state. Cell–cell signaling via diffusible factors would be a possible mechanism of action for control of the number of neurogenic progenitors.

We propose that the discovery of an ex vivo phenotype that is not maintained in vivo and the expression of multiple neuropeptides in RGCs and their receptors in neurogenic progenitor cells may point to the existence of a complex, redundant system of cell–cell signaling that modulates retinal neurogenesis. Future research could interrogate this redundant system to find a role for cell–cell signaling in development in vivo through knockout of multiple neuropeptides or receptors. Loss of function of *Vipr2* in combination with *Sstr2* might phenocopy changes in cell-type proportions similar to what is seen with perturbation of Sstr2 signaling ex vivo and is seen in vivo with loss of *Shh*. We would expect that specifically the cell types produced during the interval in which *Sstr2* and *Vipr2* are expressed would be increased in proportion relative to later-born cell types. This study demonstrates that though in vivo experiments are the gold standard for biological relevance, the lack of replication of ex vivo results in a more native context does not necessarily equate to a null result.

## Methods

### Retinal development scRNA-Seq analysis

Sequences and metadata for the mouse (accession number GSE118614) and human (GSE138002) datasets were downloaded from the Gene Expression Omnibus and used to create new Seurat objects following the Seurat SCT workflow^45,46^. Cell type classifications and ages were taken from the downloaded metadata, and used to query the expression of various neuropeptides and receptors. Correlation of neuropeptide expression and each cell type was calculated using the base R function ‘cor()’. Heatmaps were generated with R package pheatmap^47^.

### Mice

The use of animals for these studies was conducted using protocols approved by the Johns Hopkins Animal Care and Use Committee, in compliance with ARRIVE guidelines, and were performed in accordance with relevant guidelines and regulations. Timed pregnant mice were ordered from Charles River Laboratories for the smFISH experiments and embryonic retinal explant pharmacologic screen. A breeding pair of *Sstr2*^+/−^ (C57BL/6NCrl-*Sstr2*^*em1(IMPC)Mbp*^/Mmucd) mice were ordered from the Mutant Mouse Resource and Research Centers (Davis, CA) and outbred to CD1 mice. The *Sstr2*^+*/−*^ F1 progeny were bred to produce the *Sstr2*^+*/*+^, *Sstr2*^+*/−*^, and *Sstr2*^*−/−*^ mice used in this study. Similarly, two Sst^+*/−*^ male mice^48^ were received from Malcolm Low at the University of Michigan and outbred to CD1 mice. The Sst^+*/−*^ F1 progeny were then bred to produce the *Sst*^+*/*+^, *Sst*^+*/−*^, and *Sst*^*−/−*^ mice used in this study.

### Retinal sectioning

The explants and retinas used for immunostaining or smFISH were fixed in 4% paraformaldehyde in PBS rocking at room temperature for 2 h before being transferred to 30% sucrose in PBS at 4 degrees Celsius overnight. The samples were then transferred to VWR Clear Frozen Section Compound, frozen on dry ice, and stored at − 80 °C. 16 μm sections were then cut using a Leica 3050S cryostat.

### smFISH

Retinas were dissected from embryonic day (E)14 (age verified by crown-rump length) or postnatal day (P)0 mice from timed pregnant mothers. Sections were hybridized with RNAscope probes for *Sstr2* and *Neurog2* and DAPI following the RNAscope protocol. The sections were imaged on an LSM 700 confocal microscope at 20 × and 63 × resolution.

### Retinal explant culture

Retinas were dissected from E14 or E18 embryos from timed pregnant mice, age verified by crown-rump length. Dissected retinas were then flattened using a series of radial cuts, and mounted on 0.2 μm Nuclepore Track-Etch membranes on 2 ml DMEM F12, 10% FBS with 0.1% streptomycin/puromycin in 12-well plates. The explants were incubated at 37 degrees Celsius, 5% CO2 for 6 days for E14 explants, and 4 days for E18 explants. On even-numbered days, 90% of the explant culture media was replaced with new media. Treatment compounds were included in the explant culture media at the concentrations listed in the text and ordered from Tocris Bioscience.

### Explant RNA extraction and cDNA preparation

Embryonic retinal explants were removed from the nuclepore membrane and media and quick-frozen at − 80 °C or on dry ice. Total RNA was extracted from explants using the Qiagen miRNeasy Mini Kit with optional DNase digest and drying steps. RNA was extracted from 2 replicates from each condition in each of 3 rounds of experimental replicates, with a total of 6 retinas examined for each condition. The concentration of the resultant RNA was determined using a ThermoScientific NanoDrop 1000. 400 ng RNA was reversed transcribed to cDNA using the Invitrogen SuperScript IV First-Strand Synthesis System. The cDNA was diluted to 1 ng/ul in nuclease-free water.

### Explant qPCR analysis

We used the bimake.com 2 × SYBR Green qPCR Master Mix. Four ng cDNA was used for each 20 ul reaction. Three technical replicates were assayed for each sample. Samples were run on a standard 2-step amplification program on Applied Biosystems MicroAmp Fast Optical 96-Well or MicroAmp Optical 384-Well reaction plates on an Applied Biosystems StepOne Plus or ViiA 7, respectively. Primer sequences used either had been previously validated in the Blackshaw lab or were taken from the Harvard Primer Bank or generated using Primer3^40,49^. Those generated on Primer3 were required to span an exon-exon junction. All primers were verified to produce a single reaction product with traditional PCR before use for qPCR. All plots generated with ggplot2 R package^50^.

### MULTI-seq

Samples were dissociated using the Worthington papain cell dissociation system. MULTI-seq was performed to pool samples prior to 10 × Chromium Single Cell 3’ v3.0 GEM generation and library prep following the Gartner lab’s protocol using lipid-tagged oligos provided by the Gartner lab. Quality control and sequencing were performed by the Johns Hopkins Transcriptomics and Deep Sequencing Core.

### scRNAseq analysis

Sequencing results were processed through the 10 × CellRanger pipeline and the output loaded into Seurat^45^. Sample of origin for each cell was determined using the deMULTIplex R package out of the Gartner lab^40^. Seurat objects were analyzed following the Seurat SCT workflow. Cell type was determined cluster by cluster based on marker gene expression derived from the Seurat function ‘FindAllMarkers’ using default parameters^45^. This function was also used to identify differential gene expression across conditions/genotypes. Alternatively, and where noted in Results, the Seurat function ‘FindMarkers’ was used to find differential expression between two groups of cells.

Seurat objects were integrated following the Seurat Integration and Label Transfer workflow. The pseudotime trajectory was constructed and differential genes identified using the semi-supervised Monocle 2 workflow^41^. Density plots generated using ggplot2. Differential genes were clustered using the base R function ‘k-means’, and heatmap was generated using pheatmap.

### Immunostaining and visualization

Sections were stained with Invitrogen rabbit anti-Lhx2 polyclonal (1:1000), ThermoFisher rabbit monoclonal anti-Ki67 clone SP6 (1:200), or Millipore rabbit anti-Recoverin polyclonal (1:400) IgG primary antibodies, Jackson Immunoresearch Alexa-Fluor 594 donkey anti-rabbit IgG secondary antibody (1:500), and DAPI (1:5000). The stained sections were imaged on an LSM 700 confocal microscope at 20 × resolution.

Whole retinas were dissected from adult mice, flattened using a series of radial cuts, and imaged by camera next to a ruler to determine total retinal area. Area of retinas averaged for each animal.

Cell counting was performed blinded, with images randomized, using the Cell Counter plugin on ImageJ. Cells were counted in 2 200 μm long samplings and averaged for each technical replicate. Image metrics such as retinal thickness or total area were also quantified on ImageJ.

## Supplementary Information

Supplementary Information 1.

Supplementary Information 2.

## Data Availability

All sequencing data is available on GEO (GSE164741).
