# Author Correction: A potential role for somatostatin signaling in regulating retinal neurogenesis

**DOI:** 10.1038/s41598-021-96291-x

**Published:** 2021-08-16

**Authors:** Kurt Weir, Dong Won Kim, Seth Blackshaw

**Affiliations:** 1 Solomon H. Snyder Department of Neuroscience, Johns Hopkins University School of Medicine, Baltimore, MD 21205 USA; 2 Department of Ophthalmology, Johns Hopkins University School of Medicine, Baltimore, MD 21205 USA; 3 Department of Neurology, Johns Hopkins University School of Medicine, Baltimore, MD 21205 USA; 4 Institute for Cell Engineering, Johns Hopkins University School of Medicine, Baltimore, MD 21205 USA; 5 Kavli Neuroscience Discovery Institute, Johns Hopkins University School of Medicine, Baltimore, MD 21205 USA

Correction to: *Scientific Reports* 10.1038/s41598-021-90554-3, published online 26 May 2021

The original version of this Article contained an error in the Data Availability section.

“All sequencing data will be available on GEO upon publication.”

now reads:

“All sequencing data is available on GEO (GSE164741).”

The original Article has been corrected.

