# Supplementary material for: A potential role for somatostatin signaling in regulating retinal neurogenesis
Source: Sci Rep. 2021 May 26;11:10962. doi: 10.1038/s41598-021-90554-3 (PMC8155210; doi:10.1038/s41598-021-90554-3)
Supplement: Supplementary file 2 — Supplementary Information 2. [file 41598_2021_90554_MOESM2_ESM.pdf]

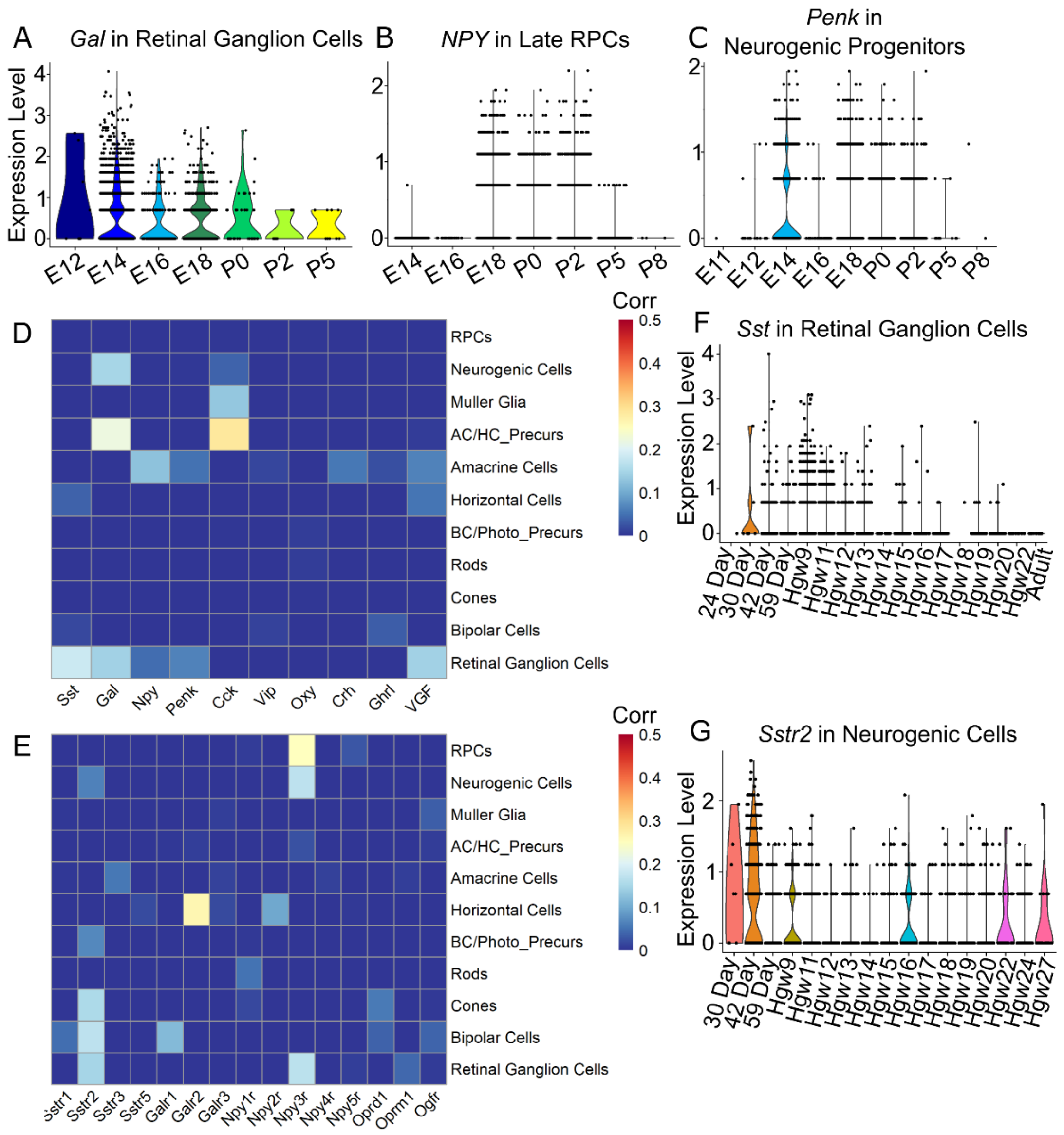

Figure S1. Cell-specific expression of neuropeptides and neuropeptide receptors in developing human retina. (A) Violin plot of expression of *Galanin* in retinal ganglion cells across development. (B) Violin plot of expression of *Neuropeptide Y* in late progenitor cells across development. (C) Violin plot of *Proenkephalin* expression in neurogenic progenitor cells across development. (D) Heatmap of correlation of expression of a neuropeptide with cell type in human retinal development. (E) Correlation of expression of a receptor with cell type in human retinal development. *SSTR4* was not expressed at detectable levels in the dataset. (F) Violin plot of expression of *SST* in retinal ganglion cells across human development. (G) Violin plot of expression of *SSTR2* in neurogenic progenitor cells in human retinal development. Correlation calculated using base R function 'cor()'.

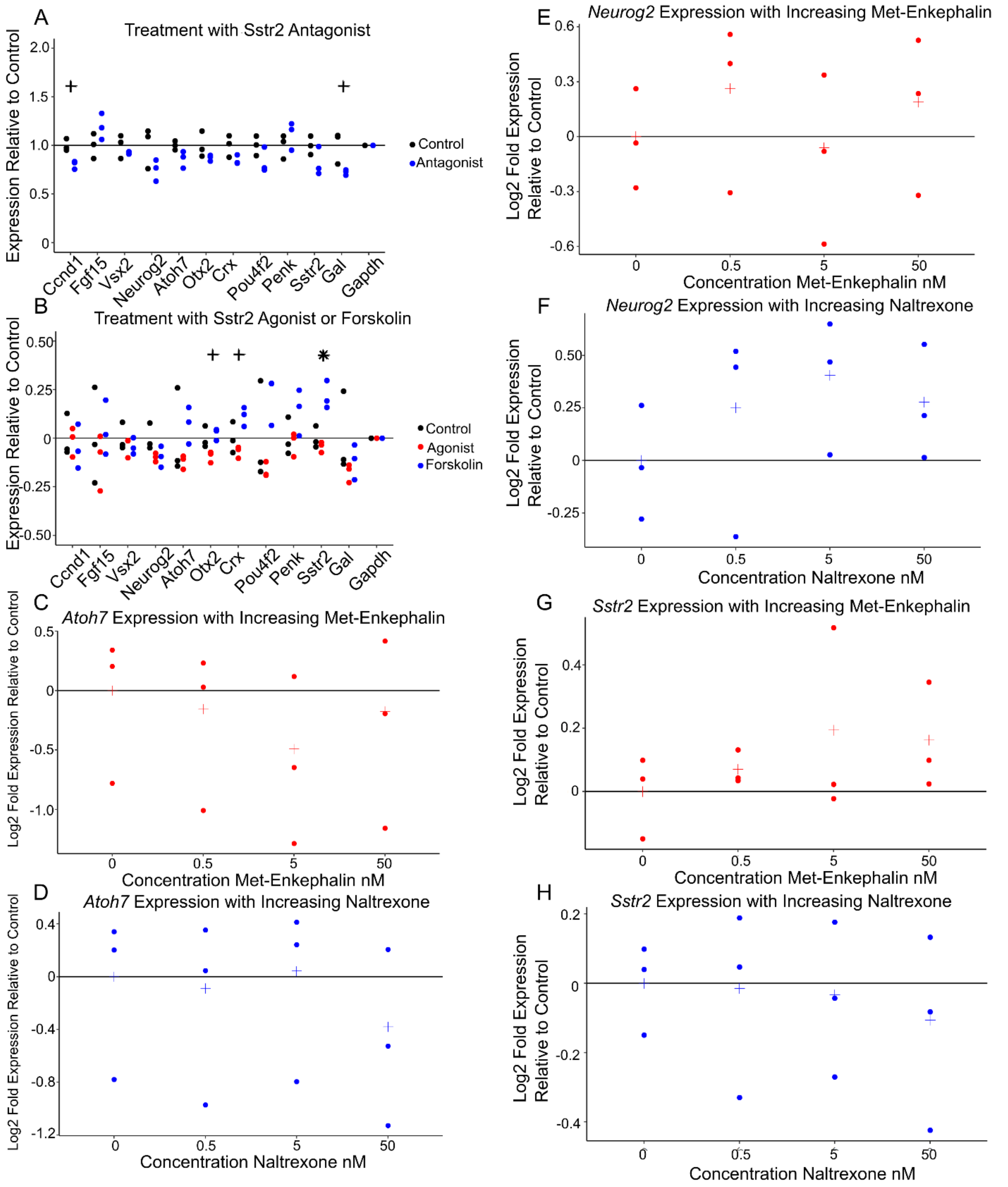

Figure S2. Treatment of embryonic retinal explants with neuropeptides, agonists and antagonists produces less impressive effects on cell type marker genes and signals. (A) Expression relative to control of marker genes for explants treated with Sstr2 antagonist CYN 154806. Concentration 1 μM. n=3 each condition. Explants treated E14-P0. (B) Marker gene expression relative to average control of explants treated with forskolin or Sstr2 agonist. Concentration 500 nM. n=3 each condition. Explants treated from E14-P0. (C) *Atoh7* expression

relative to average control of explants treated with increasing concentration of Met-Enkephalin. n=3 each condition. Explants treated E14-P0. (D) *Atoh7* expression relative to average control of explants treated with increasing concentrations of naltrexone. n=3 each condition. Explants treated E14-P0. (E) *Neurog2* expression relative to average control in explants treated with increasing concentration of Met-Enkephalin. n=3 each condition. (F) *Neurog2* expression relative to average control in explants treated with increasing concentration of naltrexone. n=3 each condition. (G) *Sstr2* expression relative to controls in explants treated with increasing concentration of Met-enkephalin. n=3 each condition. (H) *Sstr2* expression relative to average control in explants treated with increasing concentration of naltrexone. n=3 each condition. (A, B) P-value calculated using ANOVA. + indicates a nominally significant p-value below 0.05. * indicates a p-value significant at a Bonferroni correction below 0.0045.

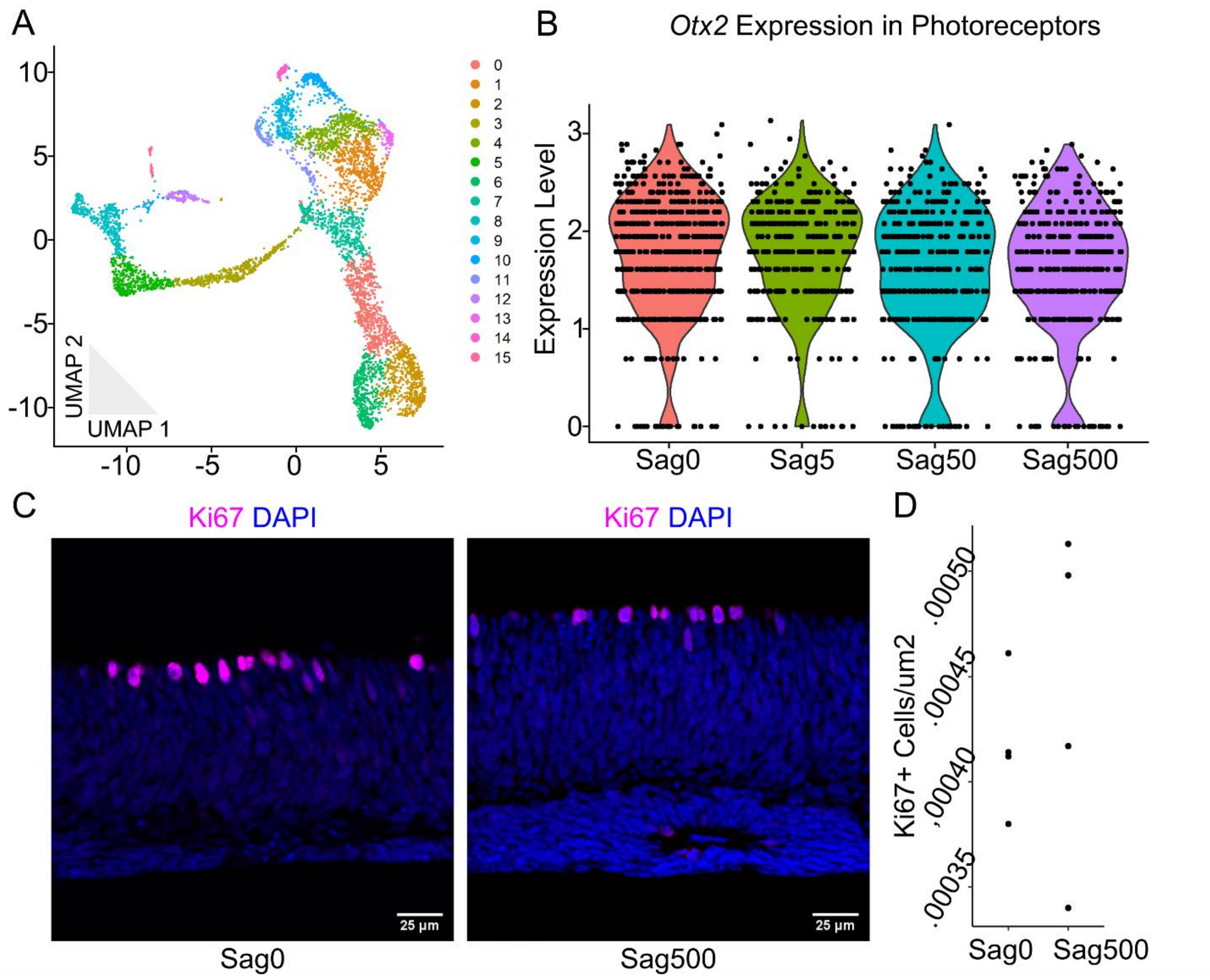

Figure S3. MULTI-seq and immunohistochemistry analysis of explants treated with four different concentrations of Sstr2 agonist reveals changing cell type proportions with increasing treatment. Four concentrations tested: 0, 5 nM, 50 nM, 500 nM Sstr2 agonist. n= 2 samples per condition (except 0 where n=1). n= 5,607 cells. (A) 2D UMAP dimension reduction representation of the pooled explant samples colored by Seurat-identified cluster. (B) Violin plot of expression of *Otx2* in photoreceptors for each condition. Was not found to be significantly different by Seurat function ‘FindAllMarkers’. (C-D) Explants stained with Ki67 antibody (magenta) and DAPI (blue) counterstaining (C) after treatment with 0 nM Sstr2 agonist (left panel) or 500 nM Sstr2 agonist (right panel). (D) Dot plot of density of Ki67+ cells for both conditions (n=4). No significant difference found by Welch’s two-sample t-test.

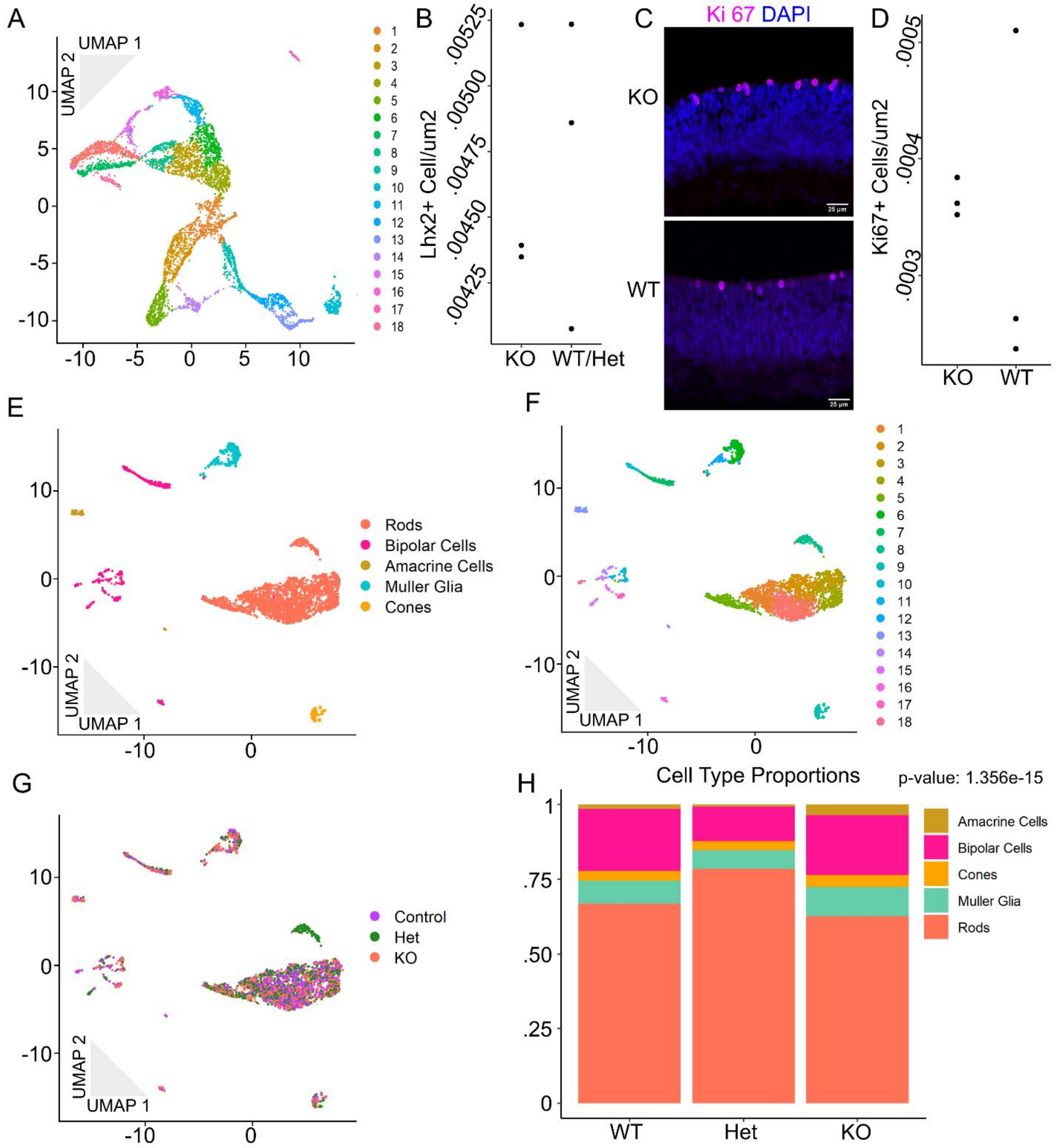

Figure S4. MULTI-seq and immunohistochemistry analysis of wildtype, *Sstr2* +/-, and *Sstr2* -/- littermates reveals no change in cell-type proportions at P0 or P14. (A) 2D UMAP dimension reduction representation of the pooled retinal cells colored by Seurat-identified cell cluster at P0. (B) Dot plot representing density of Lhx2+ cells in P0 retina (n=3). No significant difference found by Welch's two-sample t-test. (C-D) P0 retinas stained with (C) Ki67 antibody (magenta) and counterstained with DAPI (blue) with *Sstr2* -/- in the left panel and *Sstr2* +/+ in the right panel. Scale bars represent 25 µm. (D) Dot plot representing density of Ki67+ cells in P0 retina (n=3). No significant difference found by Welch's two-sample t-test. 2D UMAP dimension

reduction representation of the pooled P14 retinal cells colored by (E) cell type, (F) cluster, and (G) genotype. n= 2 samples per genotype (except wildtype where n=1). n= 3,298 cells. (H) Bar graph representation of the proportion of each cell type within each genotype. P-value for significance in difference of proportions calculated using Pearson's Chi-squared test.

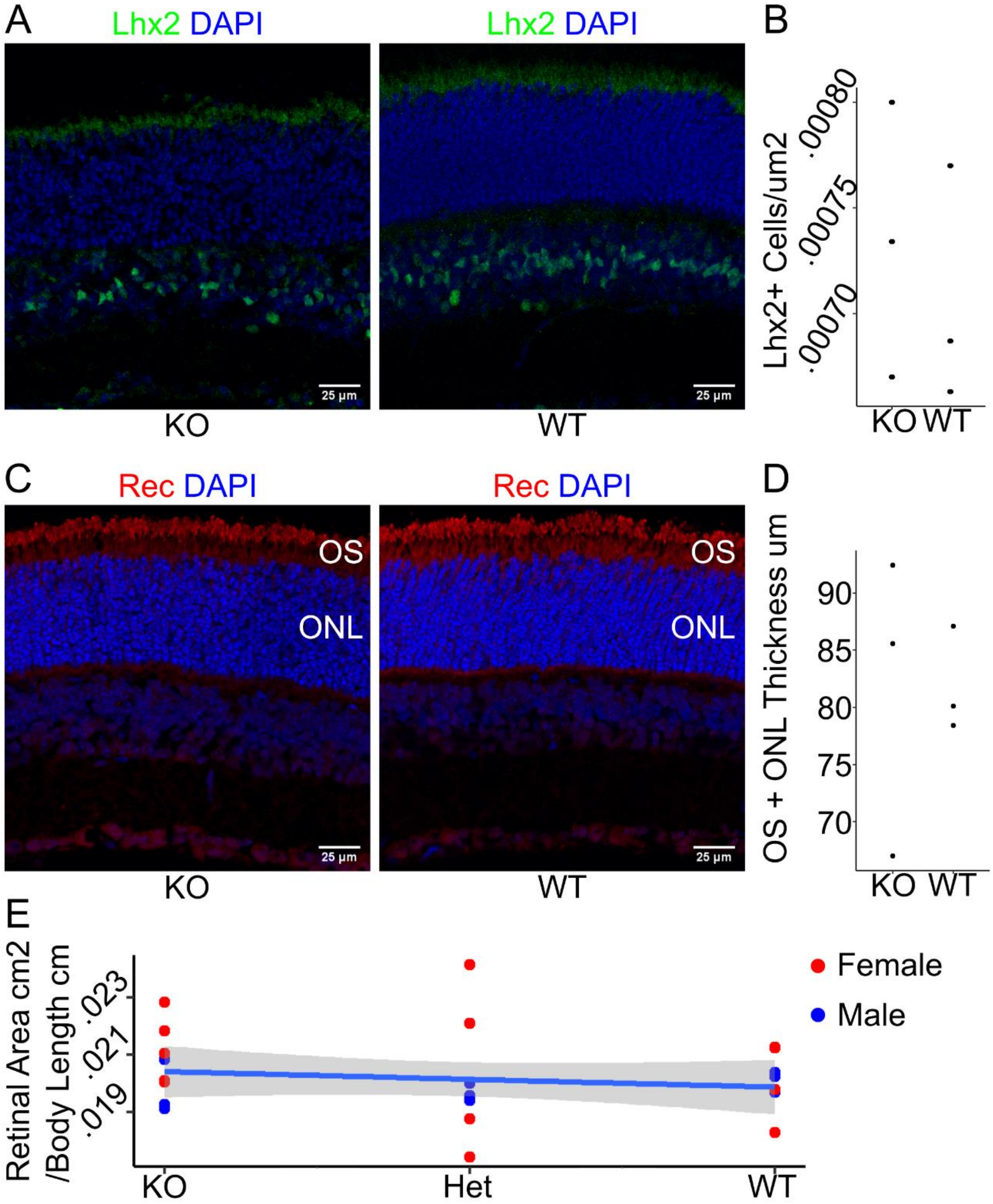

Figure S5. Immunohistochemistry analysis of P14 wildtype, *Sstr2* +/-, and *Sstr2* -/- littermates confirms no change in cell-type proportions.(A-D) Retinas stained with rabbit (A) anti-Lhx2 (green) and (C) anti-Rec (red) antibody and counterstained with DAPI (blue) with *Sstr2* -/- in the left panel and *Sstr2* +/+ in the right panel. Scale bars represent 25 µm. Dot plots representing (B) density of Lhx2+ cells and (D) combined thickness of outer segment and outer nuclear layer (n=3). No significant difference found by Welch's two-sample t-test. (E) Dot plot with fitted line and 95% confidence

interval for the line for retinal area corrected for body length across allelic series of *Sstr2* knockout. No significant impact of genotype on retinal area found by ANOVA.

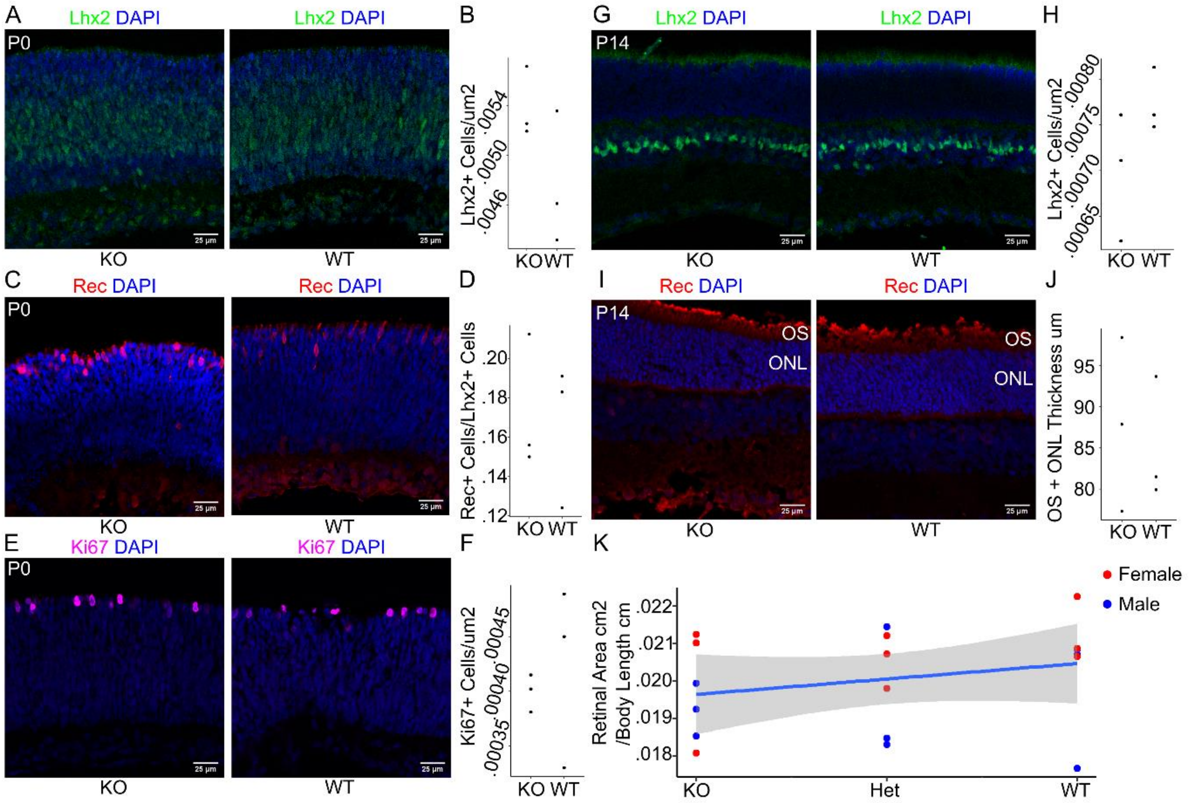

Figure S6. Immunohistochemistry analysis of P0 and P14 wildtype, *Sst* +/-, and *Sst* -/- littermates confirms no change in cell-type proportions. P0 retinas stained with rabbit (A) anti-Lhx2 (green), (C) anti-Rec (red), and (E) anti-Ki67 (magenta) antibody and counterstained with DAPI (blue). P14 retinas stained with rabbit (G) anti-Lhx2 (green) and (I) anti-Rec (red) antibody and counterstained with DAPI (blue). *Sst* -/- in the left panel and *Sst* +/+ in the right panel. Scale bars represent 25 μm. Dot plots representing (B, H) Lhx2+ cell density in P0 (B) and P14 (H), (D) Rec+ cell density corrected for Lhx2+ cell density at P0, (F) Ki67+ cell density at P0, and (J) combined outer segment and outer nuclear layer thickness at P14. No significant differences were found by Welch's two-sample t-test. (K) Dot plot with fitted line and 95% confidence interval for the line for retinal area corrected for body length across allelic series of *Sstr2* knockout. No significant impact of genotype on retinal area found by ANOVA.

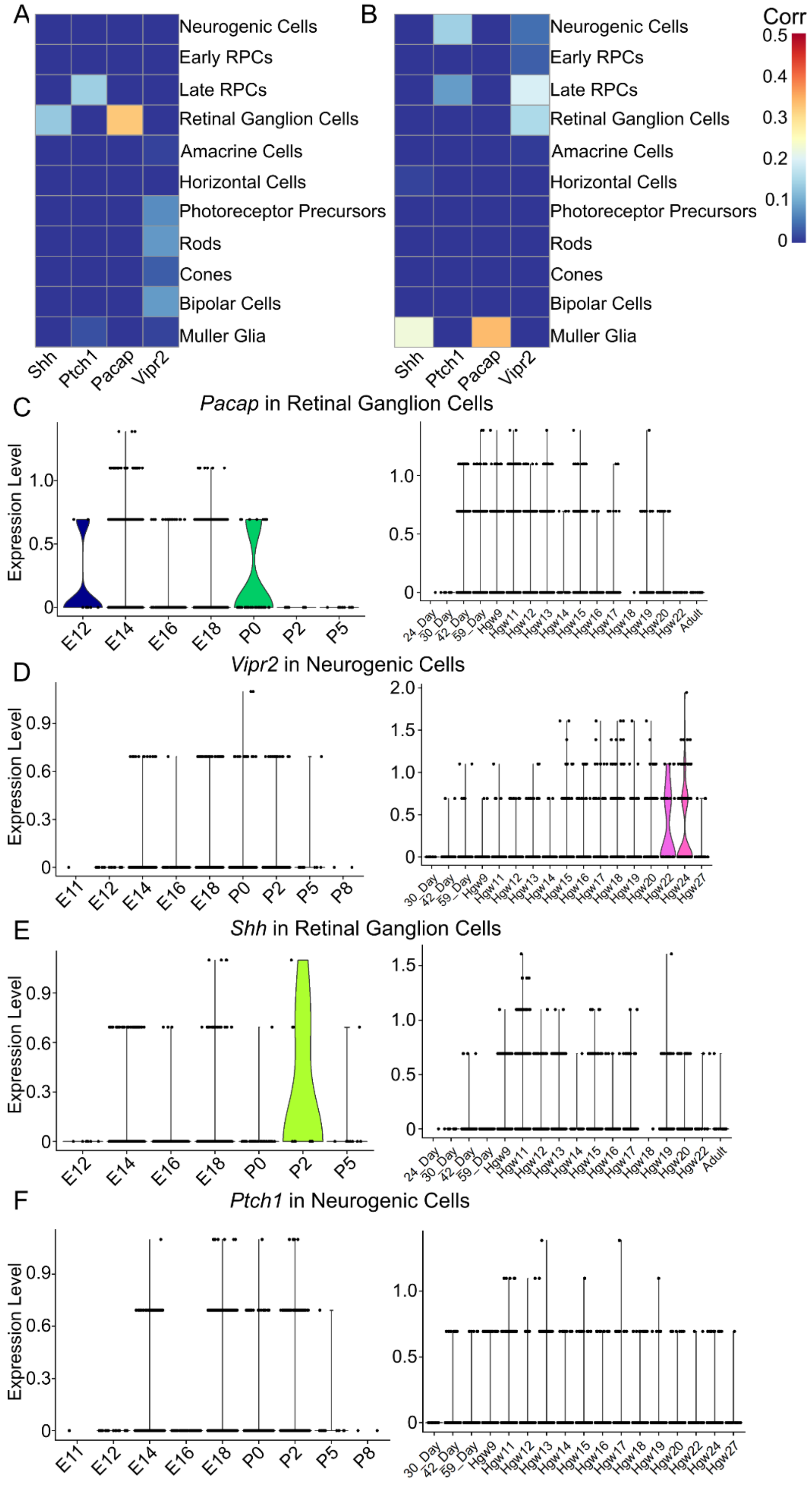

A
B
Neurogenic Cells
Early RPCs
Late RPCs
Retinal Ganglion Cells
Amacrine Cells
Horizontal Cells
Photoreceptor Precursors
Rods
Cones
Bipolar Cells
Muller Glia
Shh
Ptch1
Pacap
Vipr2
Corr
0.5
0.4
0.3
0.2
0.1
0
C
Pacap in Retinal Ganglion Cells
D
Vipr2 in Neurogenic Cells
E
Shh in Retinal Ganglion Cells
F
Ptch1 in Neurogenic Cells
Expression Level
E11
E12
E14
E16
E18
P0
P2
P5
P8
24_Day
30_Day
42_Day
59_Day
Hgw9
Hgw11
Hgw12
Hgw13
Hgw14
Hgw15
Hgw16
Hgw17
Hgw18
Hgw19
Hgw20
Hgw22
Hgw24
Hgw27
Adult

Figure S7. Additional neuropeptides show cell type and time point specificity in mouse and human retinal development. May have a redundant function with Sst. (A) Heatmap of correlation of expression of neuropeptide or receptor with cell type in mouse retinal development. (B) Heatmap of correlation of expression of neuropeptide or receptor with cell type in human retinal development. (C) Violin plots of expression of *Pacap* in retinal ganglion cells across mouse (left) and human (right) retinal development. (D) Violin plots of expression of Pacap receptor Vipr2 in neurogenic progenitor cells across mouse (left) and human (right) retinal development. (E) Violin plots of expression of ATP receptor *P2ry2* in neurogenic progenitor cells across mouse (left) and human (right) retinal development.
